# Impact of inter-reader contouring variability on textural radiomics of colorectal liver metastases

**DOI:** 10.1186/s41747-020-00189-8

**Published:** 2020-11-10

**Authors:** Francesco Rizzetto, Francesca Calderoni, Cristina De Mattia, Arianna Defeudis, Valentina Giannini, Simone Mazzetti, Lorenzo Vassallo, Silvia Ghezzi, Andrea Sartore-Bianchi, Silvia Marsoni, Salvatore Siena, Daniele Regge, Alberto Torresin, Angelo Vanzulli

**Affiliations:** 1 Department of Radiology, ASST Grande Ospedale Metropolitano Niguarda, Piazza Ospedale Maggiore 3, 20162 Milan, Italy; 2 Department of Medical Physics, ASST Grande Ospedale Metropolitano Niguarda, Piazza Ospedale Maggiore 3, 20162 Milan, Italy; 3 Department of Surgical Sciences, University of Turin, via Verdi 8, 10124 Turin, Italy; 4 Radiology Unit, Candiolo Cancer Institute, FPO-IRCCS, Strada Provinciale 142 km 3.95, 10060 Candiolo, Turin Italy; 5 Radiology Unit, SS Annunziata Hospital ASLCN1 Cuneo, via Ospedali 14, 12038 Cuneo, Savigliano Italy; 6 Niguarda Cancer Center, Grande Ospedale Metropolitano Niguarda, Piazza Ospedale Maggiore 3, 20162 Milan, Italy; 7 Department of Oncology and Hemato-Oncology, Università degli Studi di Milano, via Festa del Perdono 7, 20122 Milan, Italy; 8 Precision Oncology, IFOM – The FIRC Institute of Molecular Oncology, via Adamello 16, 20139 Milan, Italy; 9 Department of Physics, Università degli Studi di Milano, via Giovanni Celoria 16, 20133 Milan, Italy

**Keywords:** Colorectal neoplasms, Image processing (computer-assisted), Liver neoplasms, Radiomics, Tomography (x-ray, computed)

## Abstract

**Background:**

Radiomics is expected to improve the management of metastatic colorectal cancer (CRC). We aimed at evaluating the impact of liver lesion contouring as a source of variability on radiomic features (RFs).

**Methods:**

After Ethics Committee approval, 70 liver metastases in 17 CRC patients were segmented on contrast-enhanced computed tomography scans by two residents and checked by experienced radiologists. RFs from grey level co-occurrence and run length matrices were extracted from three-dimensional (3D) regions of interest (ROIs) and the largest two-dimensional (2D) ROIs. Inter-reader variability was evaluated with Dice coefficient and Hausdorff distance, whilst its impact on RFs was assessed using mean relative change (MRC) and intraclass correlation coefficient (ICC). For the main lesion of each patient, one reader also segmented a circular ROI on the same image used for the 2D ROI.

**Results:**

The best inter-reader contouring agreement was observed for 2D ROIs according to both Dice coefficient (median 0.85, interquartile range 0.78–0.89) and Hausdorff distance (0.21 mm, 0.14–0.31 mm). Comparing RF values, MRC ranged 0–752% for 2D and 0–1567% for 3D. For 24/32 RFs (75%), MRC was lower for 2D than for 3D. An ICC > 0.90 was observed for more RFs for 2D (53%) than for 3D (34%). Only 2/32 RFs (6%) showed a variability between 2D and circular ROIs higher than inter-reader variability.

**Conclusions:**

A 2D contouring approach may help mitigate overall inter-reader variability, albeit stable RFs can be extracted from both 3D and 2D segmentations of CRC liver metastases.

## Key points

- Reader contouring variability may impact on radiomic features of liver metastases from colorectal cancer (CRC).
- Stable textural features against inter-reader variability can be extracted from contrast-enhanced computed tomography images of liver metastases from CRC.
- Two-dimensional contouring seems to be less affected than three dimensional contouring by inter-reader variability.
- Two-dimensional contouring may help reduce variability of readers’ lesion segmentation.

## Background

In the current era of targeted therapies, the search for imaging biomarkers linking the genetic and molecular characteristics of tumours to the clinical and morphofunctional phenotype is pivotal to provide oncologic patients with more tailored treatment options [1, 2]. A special effort to achieve this goal is being made in colorectal cancer (CRC), one of the most common malignant tumours worldwide [3]. Since 20% of patients with CRC already have liver metastases at the time of diagnosis and up to 50% will develop them within the first 3 years [4], to improve the detection of molecular alterations over time and space of these lesions is crucial to optimise the patient’s management [5].

In this context, great expectations were raised by radiomics, namely the quantitative analysis of medical imaging for the extraction of high-throughput data with diagnostic, prognostic and predictive value [6]. Evidence correlating the textural radiomic features (RFs) extracted from the computed tomography (CT) scans of CRC liver metastases with the clinical outcomes of the patients have accumulated in the last few years. For example, texture analysis has been used to predict the tumour grade and overall survival of patients with stage IV CRC before treatment [7, 8], the response of liver metastases to first-line chemotherapy [9, 10] and the risk of liver recurrence after hepatic resection of CRC lesions [11].

However, the extraction of the RFs is a complex process with many steps, each of them characterised by specific issues that could compromise the robustness of the results [12]. Widely studied sources of uncertainty in radiomics are the image acquisition and reconstruction settings and the preprocessing manipulations [13, 14], but inter-reader variability in lesion segmentation is also critical, especially considering that the current standard of reference is manual contouring [15, 16] and that multicentric trials, involving multiple readers, are recommended to assure adequate statistical power [6, 17]. Depending on how the regions of interest (ROIs) are encompassed in the segmentation, the subsequent quantitative analysis can be significantly modified [18]. For liver metastases, this issue is particularly relevant: given that the tumour type and site are crucial aspects to consider, a higher inter-reader uncertainty is expected for lesions with blurred boundaries or low-contrast interface with the surrounding tissues [16, 19, 20]. Moreover, in terms of reproducibility and predictive value, controversies still exist regarding the choice of including in the segmented ROI the whole lesion or just its more representative cross-section [21–23].

At present, the role of contouring in RFs reproducibility has been addressed in several studies [24–27], but to the best of knowledge, none of them concerned hepatic CRC metastases. Therefore, the aim of this work is to assess the influence of inter-reader contouring variability on the texture analysis of CRC liver metastases, focusing on the role of three- and two-dimensional segmentation in determining RFs robustness. Since different approaches can impact on the results of radiomic studies but also on the time and resources needed for the data collection and analysis, to optimise the contouring strategy is essential.

## Methods

### Patients

This was an ancillary study conducted on CRC patients (*n* = 31) enrolled from 2016 to 2018 in the multicentric phase II HERACLES trial (NCT03225937), exploring the efficacy of dual human epidermal growth factor receptor 2 blockade in patients harbouring human epidermal growth factor receptor 2-amplified metastatic CRC. Clinical inclusion and exclusion criteria of the trial were previously reported [28, 29]. A further selection was performed to include only patients with liver metastases. The study was approved by the Ethics Committee, and all the patients signed written informed consent to allow the images of their diagnostic examinations to be used for scientific purposes at the time of enrolment in the study.

### Reading protocol

Two residents in the radiology department, referred as R1 for hospital 1 and R2 for hospital 2 (3 and 4 years of experience, respectively) reviewed the available imaging. For each patient, only the portal venous phase of an abdominal computed tomography (CT) scan with intravenous injection of iodinated contrast agent was used. This phase is the most used for radiomics of liver metastases and provides the best visualisation of the lesions [30].

Every metastasis was individually evaluated if suitable for the analysis (*i.e.,* to provide adequate textural information) by applying the following exclusion criteria [31, 32]: (a) maximum axial diameter lesser than 10 mm; (b) tumour boundaries not surely identifiable because of artefacts or confluent lesions. Any disagreement about the selection of specific lesions was resolved through consultation between the two readers.

Both readers measured the largest axial diameter of all the lesions with a digital calliper. The two readers contoured the whole lesion volume using a three-dimensional (3D) region of interest (ROI) and the largest and most representative area on the axial slice using a two-dimensional (2D) ROI of the metastases from the original CT images. For the segmentation task, R1 used 3D Slicer v.4.10.0 (www.slicer.org) and R2 used MIPAV (Medical Image Processing, Analysis and Visualization, http://mipav.cit.nih.gov), both of them allowing lesion contouring by generating polygonal meshes. All the sets of ROIs were exported as NIfTI binary labelmaps.

**Fig. 1:**
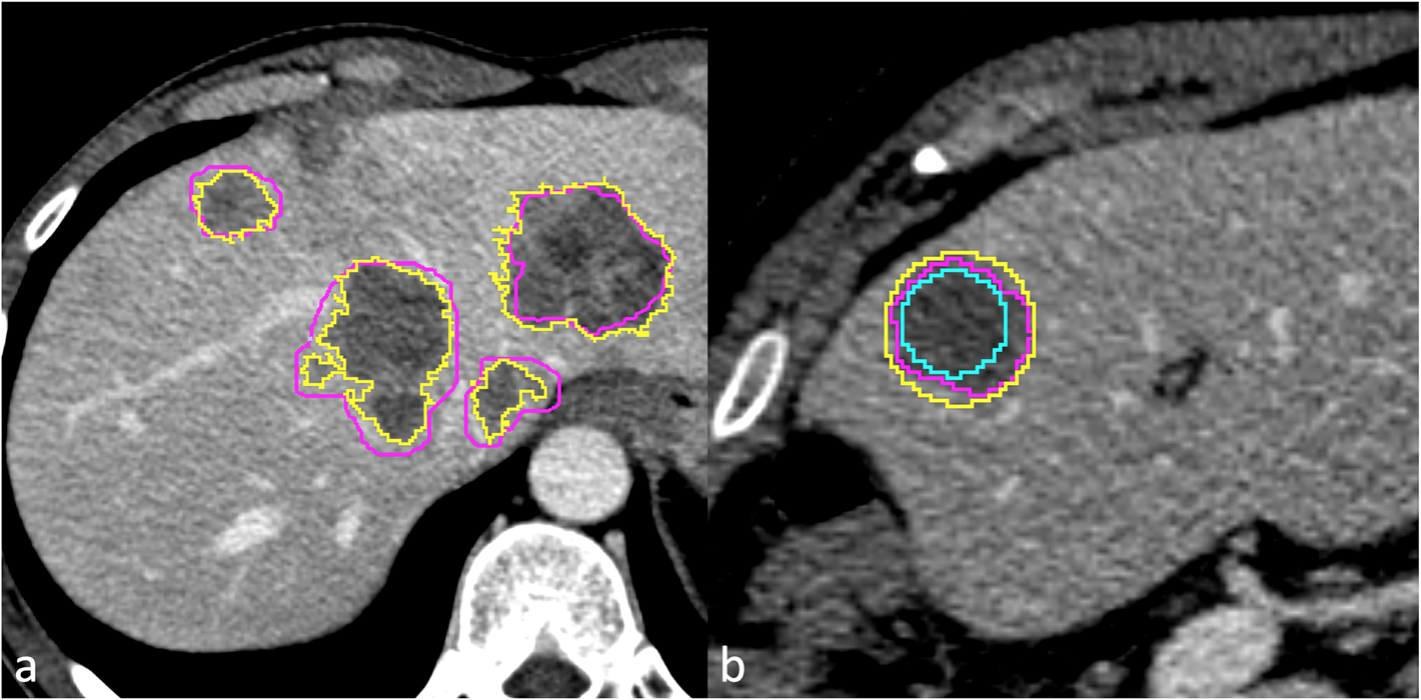
For each metastasis, the whole lesion volume and the largest axial cross-section were segmented by two readers. **a** Purple line (reader 1) *versus* yellow line (reader 2) contouring. The largest two-dimensional (2D) region of interest (ROI) of the main lesion was confronted with two circular ROIs, one inside the metastasis and one outside it. **b** Purple line (reader 1) 2D *versus* yellow line (smallest circular ROI inclusive of the whole lesion) *versus* azure line (largest circular ROI completely inside the lesion)

Considering the main lesion for each patient, in the same slice of the 2D ROI, R1 also segmented two circular ROIs (Fig. 1): the smallest inclusive of the whole metastasis and the largest one completely inside it. This additional set of segmentations was intended to assess the impact of a simplified segmentation protocol on inter-reader variability.

All segmentations were finally approved by two experienced radiologists with over 20 years of experience.

### Extraction of radiomic features

The RFs were extracted from the ROIs using Imaging Biomarker Explorer (IBEX v. 1.0β) platform, a free open-source software developed by the MD Anderson Cancer Center (Houston, USA). For this study, 32 textural RFs from the *grey level co-occurrence matrix* (GLCM) and the grey level run length matrix (GLRLM) were considered [33]. Also, max 3D diameter, number of voxels, and volume were extracted from the ROIs. For the calculated RFs, the consistency with the image biomarker standardisation initiative (IBSI) standard was verified using an IBSI-validated in-house developed software [34].

The RF extraction in the study was performed on the original CT image setting in IBEX a fixed range of 480 grey levels (from -200 to 279 HU) discretised in 32 bins, with offset = 1 and symmetry = 1. All the directions allowed by the software were considered. The range was reasoned on the grey level distribution of the overall metastases, whilst the number of bins was chosen as a compromise to limit the noise contribution and the loss of texture information [35].

No resampling nor other preprocessing were applied to the CT images.

### Data analysis

All analyses were performed on Microsoft® Office Excel spreadsheet, except for the calculation of the intraclass correlation coefficient (ICC) based on a single-reader two-way random-effects model, which was performed on R v.3.5.1 (“Psych” package). When required, statistical significance was established at the *p* < 0.050 level.

The inter-reader contouring agreement on both 3D and 2D ROIs was evaluated through two similarity indices: average Hausdorff distance (HD) and Dice coefficient (DC), both calculated with “SlicerRT” toolkit [36]. Hausdorff distance measures how far two subsets of a metric space are from each other, thus indicating the longest distance between the boundaries of two contours. The average HD was chosen so as to have a better representation of global contouring discrepancy [37]. Instead, the DC quantifies the spatial overlap between two contours/volumes, ranging from 0 for null overlapping and 1 for perfect overlapping [38]. The two indices emphasise different characteristics: the DC quantifies the discrepancy in voxel labelling, whilst the HD performs better at detecting deviations (spikes, holes, etc.) which alter the contour shape but do not substantially modify the volume [26].

For both DC and HD, calculated over all the segmentations, median value and interquartile range (IQR) were reported. The Wilcoxon signed-rank test was performed to evaluate if there was a significant difference between the indices calculated for 2D and 3D ROIs. Spearman’s *rho* correlation coefficient between the values of DC and HD was also calculated.

To verify if the size of the metastases could influence the inter-reader agreement, a linear regression analysis was performed to evaluate the association between the following parameters: manual largest axial diameter and max 3D diameter *versus* DC and HD; volume/area (cm^3^/cm^2^) *versus* DC and HD. The strength of correlation was reported following Evans’s interpretation [39].

The influence of inter-reader variability on the extracted RFs was assessed considering the relative change [(RF_R1_ −  RF_R2_)/RF_R1_)] both on 3D and 2D ROIs for all lesions. For each RF, the mean relative change (MRC) was calculated. The inter-reader MRC in RF values was also compared with the MRC obtained from R1 2D ROIs against the circular ROIs, taking R1 values as a reference.

The ICC of the RFs between the two readers was calculated to describe how strongly the two datasets resembled each other and so to guide the selection of RFs according to robustness [40]. In accordance with the literature [41], the ICC was interpreted as follows: poor agreement for ICC ≤ 0.50; moderate agreement for 0.50 < ICC ≤ 0.75; good agreement for 0.75 < ICC ≤ 0.90; excellent agreement for ICC > 0.90.

## Results

Of the original 31 patients of the trial, 14 (45%) were excluded because they had no liver metastases (*n* = 6), because of the presence of imaging artefacts (*n* = 3) or because there were only lesions < 10 mm (*n* = 2) or only confluent metastases largely occupying the liver parenchyma (*n* = 3). Therefore, 17 patients from 3 different centres were finally included and, according to the selection criteria, a total of 70 lesions were considered suitable for the analysis by the readers. The demographical data of the included patients are reported in Table 1, whilst detailed information about the acquisition and reconstruction parameters of their CT scans are listed in Table 2.

**Table 1:** Demographical data and number of analysed metastases for each patient enrolled in the study

Patient	Age at CT (years)	Sex	Primary cancer site	Number of analysed metastases	Lines of treatment	Chemotherapy regimens
1	77	F	Rectum	1	2	FOLFOX + bevacizumab FOLFIRI + cetuximab
2	66	M	Colon (left)	5	2	FOLFOX FOLFIRI + cetuximab
3	62	M	Colon (left)	3	4	FOLFIRI + cetuximab Regorafenib Trifluridine/tipiracil Capecitabine
4	59	M	Colon (left)	1	2	FOLFOX FOLFIRI + cetuximab
5	56	M	Rectum	1	2	FOLFIRI + cetuximab Not available
6	40	M	Colon (left)	2	2	FOLFOX + panitumumab FOLFIR I + bevacizumab
7	61	M	Colon (left)	4	2	FOLFOX + panitumumab FOLFIRI + aflibercept
8	56	M	Colon (right)	6	2	FOLFOX + panitumumab FOLFIRI + aflibercept
9	47	M	Colon (left)	5	2	FOLFOX + cetuximab FOLFIRI + bevacizumab
10	32	M	Colon (left)	7	3	FOLFOXIRI + bevacizumab FOLFIRI + aflibercept Panitumumab
11	66	M	Colon (left)	6	2	XELIRI FOLFOX + bevacizumab
12	63	F	Colon (left)	8	2	FOLFOX + panitumumab FOLFIRI + bevacizumab
13	61	F	Colon (left)	1	2	XELOX + bevacizumab FOLFIRI + cetuximab
14	52	M	Rectum	2	2	FOLFOX FOLFOX + bevacizumab
15	41	M	Colon (left)	5	1	FOLFOX + panitumumab
16	59	M	Rectum	4	1	FOLFIRI + bevacizumab
17	60	M	Colon (left)	9	3	FOLFOX + cetuximab FOLFIRI + bevacizumab FOLFIRI

Previous drug regimens are reported in chronological order of administration. All patients had histological-confirmed adenocarcinoma of the colon/rectum with metastatic liver disease not amenable to salvage surgery. In all cases, the primary tumour was KRAS (Kirsten rat sarcoma) wild-type and HER2 (human epidermal growth factor 2) positive

*F* Female, *M* Male, *FOLFIRI* Leucovorin + fluorouracil + irinotecan, *FOLFOX* Leucovorin + fluorouracil + oxaliplatin, *FOLFOXIRI* Leucovorin + fluorouracil + oxaliplatin + irinotecan, *XELIRI* Capecitabine + irinotecan, *XELOX* Capecitabine + oxaliplatin

**Table 2:** Acquisition and reconstruction parameters extracted from the header DICOM of the computed tomography scans, patient by patient

Patient	Manufacturer	Model	Slice thickness (mm)	Increment (mm)	Pixel size (mm^2^)	kVp	Kernel
1	Siemens	Sensation 64	3	3	0.8242 × 0.8242	120	B30f
2	Siemens	Somatom Definition	3	3	0.7031 × 0.7031	120	B30f
3	Philips	Brilliance 64	3	3	0.8730 × 0.8730	100	B
4	Siemens	Sensation 64	3	3	0.7812 × 0.7812	120	B30f
5	Siemens	Sensation 64	3	3	0.8750 × 0.8750	120	B30f
6	Toshiba	Aquilion	3	3	0.7210 × 0.7210	120	FC13
7	Siemens	Sensation 64	3	3	0.7852 × 0.7852	120	B30f
8	Siemens	Sensation 64	3	3	0.8047 × 0.8047	120	B30f
9	Siemens	Somatom Definition	3	3	0.7773 × 0.7773	100	B30f
10	Hitachi	Eclos	2.5	2.5	0.7100 × 0.7100	120	32
11	Hitachi	Eclos	2.5	2.5	0.8410 × 0.8410	120	32
12	Siemens	Somatom Definition	3	2	0.6875 × 0.6875	100	I30f/3
13	GE	Optima CT520 Series	2.5	2.5	0.8477 × 0.8477	120	Standard
14	GE	LightSpeed Pro 32	0.625	0.625	0.8926 × 0.8926	120	Standard
15	Siemens	Somatom Definition	3	2	0.6328 × 0.6328	100	I30f/3
16	Siemens	Somatom Definition	3	2	0.7969 × 0.7969	100	I30f/3
17	Siemens	Somatom Definition	3	2.5	0.7344 × 0.7344	120	I30f/3

All images had a matrix size of 512 × 512 and were acquired 70–80 s after contrast injection with an automatic exposure control system

The largest axial diameter of the selected lesions ranged from 10 to 80 mm, with a median value of 27 mm (IQR 17–29 mm) according to R1 and 26 mm (IQR 16–26 mm) according to R2.

### Contouring variability

**Fig. 2:**
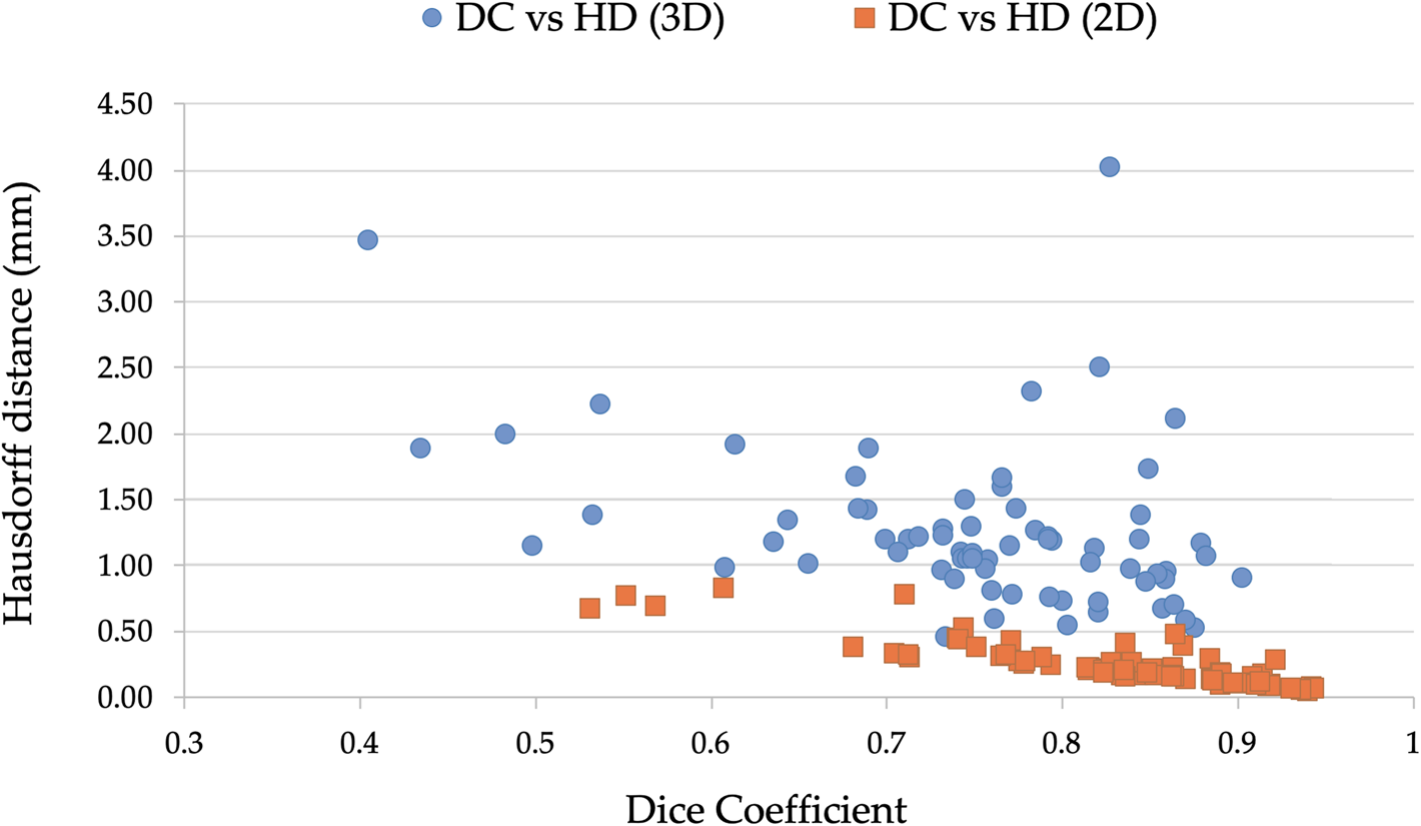
Correlation between Dice coefficient and average Hausdorff distance calculated for the two-dimensional (2D) and three-dimensional (3D) regions of interest (ROIs) segmented by reader 1 and reader 2. 2D ROIs, Spearman *rho* = -0.85 (*p* < 0.001); 3D ROIs, Spearman *rho* = -0.38 (*p* < 0.001)

**Fig. 3:**
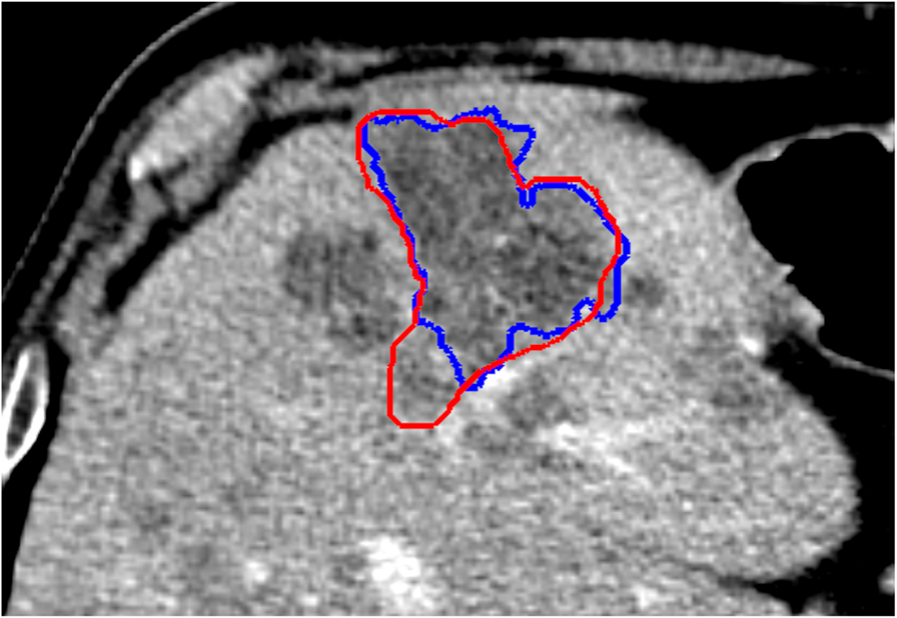
Example of discrepancy between similarity indices (patient number 7): Dice coefficient was 0.86 (median two-dimensional, 0.85), whilst average Hausdorff distance was 0.48 mm (median two-dimensional, 0.21 mm). The regions of interest (blue and red lines) were approximately overlapping, but the readers differently interpreted the nature of a hypodense area adjacent to the metastasis

Moving from 3D to 2D ROIs, an increase in DC and a reduction in HD was observed. Specifically, 3D ROIs showed a median DC of 0.76 (IQR 0.71–0.82) and a median HD of 1.15 mm (IQR 0.90–1.41 mm). For 2D ROIs, the median DC was of 0.85 (IQR 0.78–0.89), and median HD was of 0.21 mm (IQR 0.14–0.31 mm). According to Wilcoxon signed-rank test, these differences were significant for both DC (*p* < 0.001) and HD (*p* < 0.001). Moreover, a very strong negative correlation was found between HD and DC for 2D ROIs (*rho* = -0.85; *p* < 0.001), but only a weak negative correlation was found for 3D ones (*rho* = -0.38; *p* < 0.001) (Fig. 2). An example of discrepancy between the two similarity indices is presented in Fig. 3.

Weak-to-moderate correlations (-0.45 ≤ *rho* ≤ 0.45) were found between the two similarity indices and size parameters for both 3D and 2D ROIs (Table 3).

**Table 3:** Correlation results (Spearman’s *rho* coefficients) between similarity indices (Dice coefficient and average Hausdorff distance) and size parameters of the segmented metastases for both 2D and 3D ROIs

Correlation	2D ROI		3D ROI	
	DC	HD	DC	HD
ROI manual axial diameter	0.42 (*p* < 0.001)	-0.04 (*p* = 0.760)	0.45 (*p* < 0.001)	0.45 (*p* < 0.001)
ROI maximum 3D diameter	0.37 (*p* < 0.001)	-0.12 (*p* = 0.382)	0.42 (*p* < 0.001)	0.41 (*p* = 0.002)
ROI volume/area	0.30 (*p* < 0.001)	-0.17 (*p* = 0.209)	0.36 (*p* < 0.001)	0.30 (*p* = 0.024)

*2D* Two dimensional, *3D* Three dimensional, *DC* Dice coefficient, *HD* Hausdorff distance (average), *ROI* Region of interest

### Impact on texture analysis

**Fig. 4:**
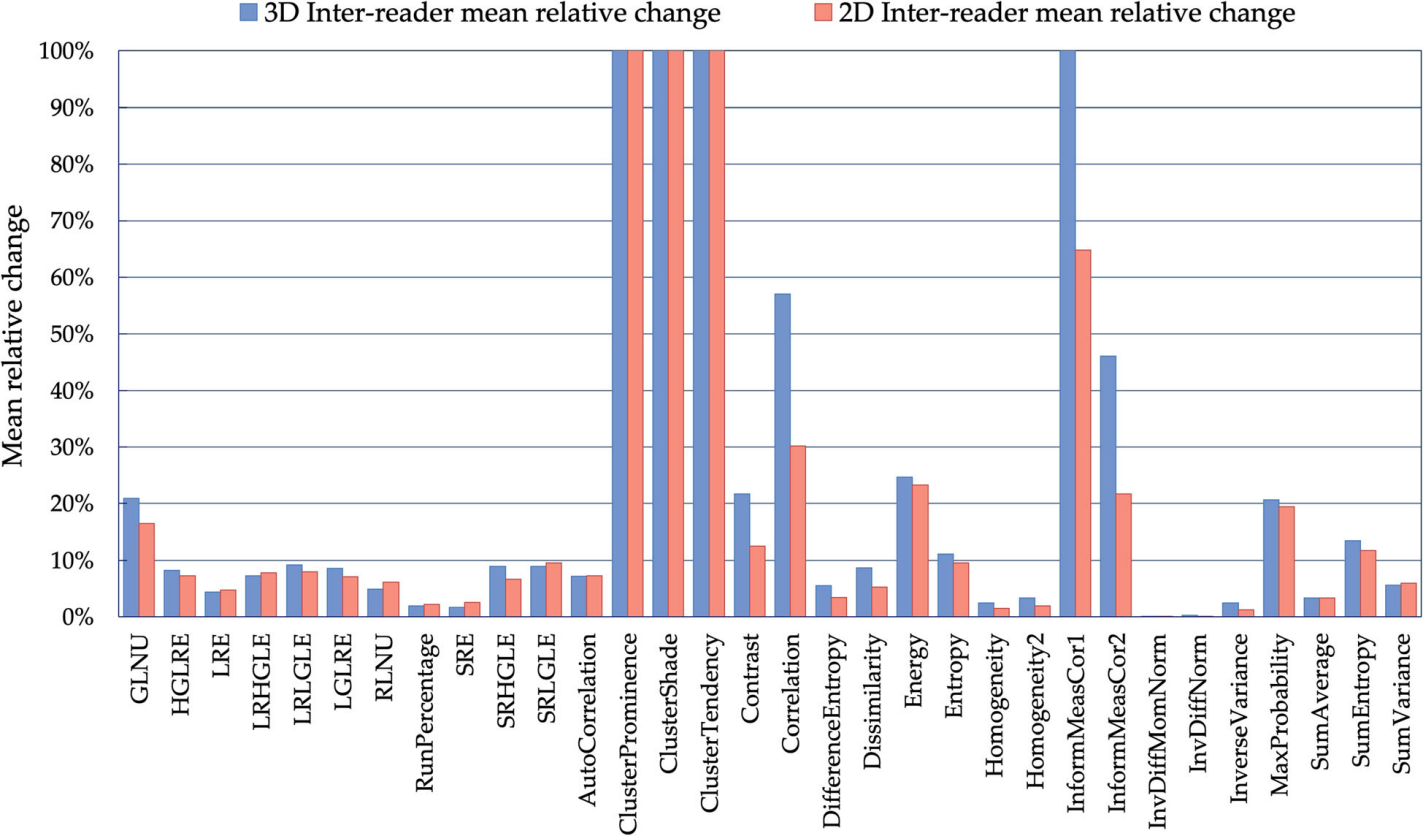
Means of relative changes between the RFs extracted from each lesion (*n* = 70) contoured by the two readers. The results from two-dimensional and three-dimensional segmentations were compared. Out of scale values have been truncated. The cluster features showed the greatest instability between readers. *GLNU* Grey level non-uniformity, *HGLRE* High grey level run emphasis, *LGLRE* Low grey level run emphasis, *LRE* Long run emphasis, *LRHGLE* Long run high grey level emphasis, *LRLGLE* Long run low grey level emphasis, *RLNU* Run length non-uniformity, *SRE* Short run emphasis, *SRHGLE* Short run high grey level emphasis, *SRLGLE* Short run low grey level emphasis

As illustrated in Fig. 4, different RFs showed to have different susceptibility to inter-reader variability. In particular, inter-reader MRC ranged from 0 to 1567% for 3D ROIs and from 0 to 752 for 2D ROIs. The inter-reader discrepancy was below 10% for more than 60% of the RFs extracted from both the sets of ROIs. For 24/32 (75%) RFs, the discrepancies were lower when calculated from 2D than 3D ROIs. Specifically, this applied to 5/11 (45%) of GLRLM RFs and to 19/21 (90%) of GLCM RFs.

**Fig. 5:**
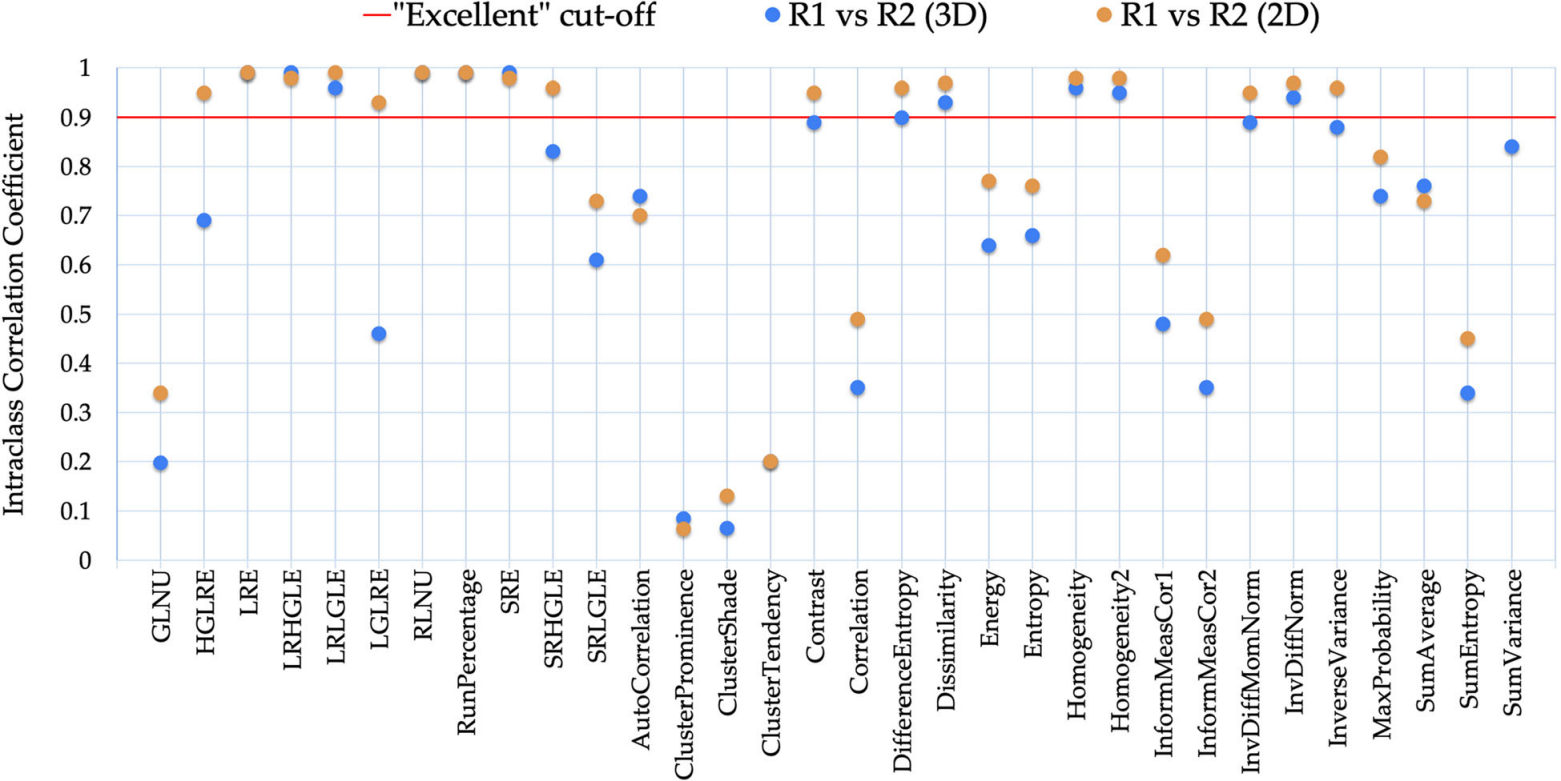
For all radiomic features, the intraclass correlation coefficients (ICC) of inter-reader variability are plotted and compared between three-dimensional and two-dimensional segmentations. “Excellent” ICC cutoff is shown as a red line. *R1* Reader 1, *R2* Reader 2, *GLNU* Grey level non-uniformity, *HGLRE* High grey level run emphasis, *LGLRE* Low grey level run emphasis, *LRE* Long run emphasis, *LRHGLE* Long run high grey level emphasis, *LRLGLE* Long run low grey level emphasis, *RLNU* Run length non-uniformity, *SRE* Short run emphasis, *SRHGLE* Short run high grey level emphasis, *SRLGLE* Short run low grey level emphasis

The ICC gave similar results in terms of RF robustness (Fig. 5). In particular, 11/32 (34%) RFs for 3D ROIs and 17/32 (53%) RFs for 2D ROIs were found to be very robust (ICC > 0.90). In both cases, the ICC of inter-reader variability ranged from 0.06 to 0.99.

When combining the results from MRC and ICC analysis (Table 4), the following RFs were found most stable: long run emphasis, long run high grey level emphasis, low grey level run emphasis, run percentage, and short run emphasis= for GLRLM as well as difference entropy, dissimilarity, homogeneity 1, homogeneity 2, and inverse difference normalised for GLCM.

**Table 4:** Mean relative changes and intraclass correlation coefficients are reported for all the textural features and both the 2D and 3D ROI sets

		2D ROI		3D ROI	
		Relative change	ICC	Relative change	ICC
GLRLM	GLNU	16%	0.34	21%	0.20
	HGLRE	7%	0.95	8%	0.69
	**LRE**	**5%**	**0.99**	**4%**	**0.99**
	**LRHGLE**	**8%**	**0.98**	**7%**	**0.99**
	LRLGLE	**8%**	**0.99**	**9%**	**0.96**
	**LGLRE**	7%	0.93	9%	0.46
	RLNU	**6%**	**0.99**	**5%**	**0.99**
	**Run percentage**	**2%**	**0.99**	**2%**	**0.99**
	**SRE**	**3%**	**0.98**	**2%**	**0.99**
	SRHGLE	7%	0.96	9%	0.83
	SRLGLE	10%	0.73	9%	0.61
GLCM	AutoCorrelation	7%	0.70	7%	0.74
	Cluster prominence	418%	0.06	641%	0.08
	ClusterShade	752%	0.13	1567%	0.06
	Cluster tendency	100%	0.20	116%	0.20
	Contrast	13%	0.95	22%	0.89
	Correlation	30%	0.49	57%	0.35
	**Difference entropy**	**3%**	**0.96**	**6%**	**0.90**
	**Dissimilarity**	**5%**	**0.97**	**9%**	**0.93**
	Energy	23%	0.77	25%	0.64
	Entropy	10%	0.76	11%	0.66
	**Homogeneity**	**1%**	**0.98**	**2%**	**0.96**
	**Homogeneity2**	**2%**	**0.98**	**3%**	**0.95**
	InformationMeasureCorrel1	65%	0.62	136%	0.48
	InformationMeasureCorrel2	22%	0.49	46%	0.35
	InverseDifferMomentNormal	0%	0.95	0%	0.89
	**InverseDifferNormal**	**0%**	**0.97**	**0%**	**0.94**
	InverseVariance	1%	0.96	2%	0.88
	MaxProbability	19%	0.82	21%	0.74
	SumAverage	3%	0.73	3%	0.76
	SumEntropy	12%	0.45	14%	0.34
	SumVariance	6%	0.80	6%	0.84

Bold text is used for features found robust against inter-reader variability (ICC > 0.90 and mean relative change < 10%)

*2D* Two dimensional, *3D* Three dimensional, *GLCM* Grey level co-occurrence matrix, *GLNU* Grey level non-uniformity, *GLRLM* Grey level run length matrix, *HGLRE* High grey level run emphasis, *ICC* Intraclass correlation coefficient, *LGLRE* Low grey level run emphasis, *LRE* Long run emphasis, *LRHGLE* Long run high grey level emphasis, *LRLGLE* Long run low grey level emphasis, *RLNU* Run length non-uniformity, *SRE* Short run emphasis, *SRHGLE* Short run high grey level emphasis, *SRLGLE* Short run low grey level emphasis.

Comparing RF values from R1 2D ROIs with those from the circular ROIs, a lower discrepancy between R1 and R2 ROIs was observed in most cases (Table 5). In particular, taking account of the MRC, inter-reader variability was equal or preponderant for 30/32 (94%) RFs.

**Table 5:**
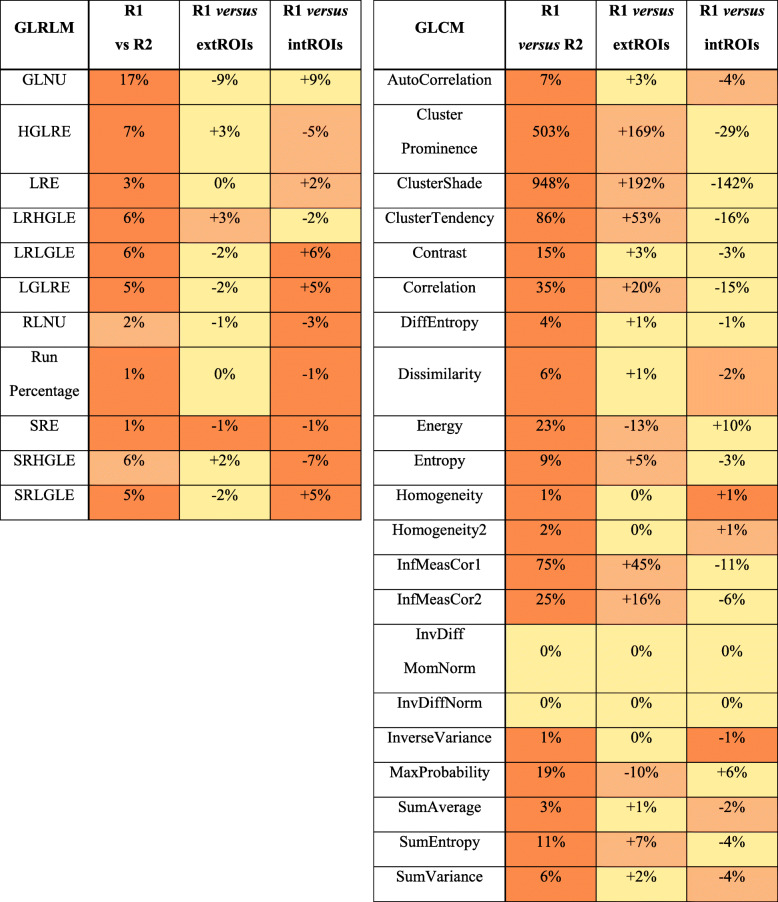
Comparison of the radiomic features obtained using manual 2D ROIs by R1 *versus* R2 and, for R1, using manual *versus* circular 2D ROIs

For the main lesion of each patient, the RFs from R1 2D ROIs were compared to R2 2D ROIs and to the circular 2D ROIs (extROIs and intROIs). Mean relative discrepancy taking R1 values as reference is reported. Inter-reader variability was preponderant for nearly all RFs. As expected, RFs from extROIs and intROIs had a divergent behaviour in respect of R1 2D ROIs, whose characteristics were intermediate. The colour code refers to the absolute value of discrepancy

*2D* Two-dimensional, *extROI* Smallest circular segmentation including the whole lesion, *GLCM* Grey level co-occurrence matrix, *GLNU* Grey level non-uniformity, *GLRLM* Grey level run length matrix, *HGLRE* High grey level run emphasis, *intROI* Largest circular segmentation completely inside the lesion, *LGLRE* Low grey level run emphasis, *LRE* Long run emphasis, *LRHGLE* Long run high grey level emphasis, *LRLGLE* Long run low grey level emphasis, *R1* Reader 1, *R2* Reader 2, *RFs* Radiomic features, *RLNU* Run length non-uniformity, *ROIs* Region of interest, *SRE* Short run emphasis, *SRHGLE* Short run high grey level emphasis, *SRLGLE* Short run low grey level emphasis

## Discussion

In this study, the impact of inter-reader contouring variability on texture analysis of CRC liver metastases was assessed comparing the 3D and 2D ROIs of 70 lesions from 17 patients and the respectively extracted RFs.

The segmentation process of liver metastases is a challenging task due to the site and the vague boundaries of the lesions. However, we obtained satisfactory mean DC values, consistent with similar studies [19, 42, 43]. Also, as suggested by the weak correlation between the similarity indices and the lesion size, the influence of the latter on segmentation variability seemed limited.

In general, the inter-reader contouring agreement was significantly better for 2D ROIs rather than 3D ROIs. As far as the latter set, considering that HD is more sensitive to ROI shape variation than DC [26], pairs of segmentations with high values for both the similarity indices were more common. Indeed, in 3D volume segmentation, the more peripheral slices along the *z*-axis containing the lesion suffer more for partial volume effect and the impact of all the sources of variability is greater [44, 45]. The median values of the two similarity indices and the correlation found between them for the 2D ROIs corroborated this finding.

The improvement in contouring agreement observed for 2D ROIs predictably corresponded to a reduction of inter-reader discrepancy for the majority of the RFs, although as small as the number of RFs robust to inter-reader variability was similar in the 3D and 2D sets. The robustness of these RFs was confirmed also by the ICC, so that there was correspondence between RFs with low inter-reader variability and RFs with a good or excellent ICC.

Analysing the RFs with the greatest instability, it is reasonable to believe that mathematical issues, like the high exponents (*e.g.,* power 3 or 4) in the formula of the “cluster” features, contribute to amplify the differences in the ROIs. On the other hand, the RFs most influenced by contouring variability may also be the most sensitive ones to texture variation, *i.e.,* those with the best capability to capture the information within the CT images of CRC liver metastases, and thus conceivably, the RFs with the best potential predictive value. For example, Simpson et al. [11] found that “contrast, correlation and homogeneity” were associated with hepatic disease-free survival in patients with CRC liver metastases. In the current analysis, the first two RFs showed a mild-to-high inter-reader variability, which is consistent with a greater sensitivity to texture variation.

These aspects must be considered when choosing the RFs to create radiomics predictive models since the “noise” related to inter-reader variability could eclipse meaningful information in the texture of CRC liver metastases, but the selection of only very robust RFs may be inadequate to detect differences in the image texture as well.

The ideal solution to eliminate the interference of inter-reader variability would be to dispose of semiautomatic or, preferably, automatic methods for the segmentation of liver metastases [15, 26]. However, the tools currently available are not yet reliable enough, as shown by testing 24 valid state-of-the-art liver tumour segmentation algorithms [43], so that operator input remains indispensable [46].

Interestingly, as shown by the comparison between standard ROIs and circular ROIs, when one of the readers drew simple geometric ROIs, less tailored on the lesion boundaries, the discrepancy in RFs values were lower or comparable to that relative to the other reader. This suggests that in the multicentric setting inter-reader variability may be handled in two ways: involving a large number of readers, so as to allow the selection of robust RFs according to individual reproducibility (*e.g.,* including RFs with ICC > 0.90 in final models) [47]; or with a “centralised” approach based on few readers to minimise variability. In the second case, a simplified segmentation protocol to accelerate the contouring task could be followed, as it would introduce a variability at most equivalent to that determined by multiple readers.

However, such analysis was limited to the 2D ROIs due to the complexity of applying it to the 3D ones, so it should be verified with larger samples. A viable compromise between assessing the lesion in its entirety and limiting the inter-reader disagreement could be to exclude from the segmentation the most peripheral slices along the Z-axis of the metastasis. Alternatively, clinical radiomic-based models could mix RFs extracted from 3D and 2D ROIs on the basis of their dependency on inter-reader variability, provided that the selection and extraction of the 2D ROIs may require additional work unless implementing automatic processes.

These methods are worthy of future investigation, considering that the main limitation of our study is not being able to assess how the improvement of RFs stability against contouring variability impact on the predictive performance due to cohort size. Indeed, only few patients were assessed, but each metastasis was singularly considered, so that the number of lesions analysed was consistent with similar works. Another limitation is that the impact of the acquisition/reconstruction settings of CT scans was not considered. The heterogeneity of scanning equipment and protocols, due to the time span and referral of patients from different institutions, could have reduced the congruency of the segmentation, but this rather strengthens the results about the textural features found to be stable. Also, two different contouring softwares were used, although eventual differences hence derived can be considered incorporable in the concept of inter-reader variability itself and, in general, it better replicated a likely situation in multicentric settings. Finally, the study focused only on the second-order features.

In conclusion, the current study highlighted the possibility to extract textural RFs robust against contouring variability from CRC liver metastases. This is essential to translate radiomics into clinical practice since the creation of large labelled imaging datasets will necessarily require the involvement of multiple readers. For the most stable RFs, both 3D and 2D segmentations were reliable, but a 2D approach, which is more pragmatic and less time-consuming, could mitigate inter-reader contouring variability. This may expand the choice of RFs suitable for building clinical models, but further studies evaluating the relationship between segmentation strategy and outcome predictivity are warranted, so as to optimise the extraction of meaningful information from the CT texture of CRC liver metastases.

## Data Availability

The datasets generated and/or analysed during the current study are not publicly available because of the terms of the research participant consent but are available from the corresponding author on reasonable request.

## Abbreviations

CRC: Colorectal cancer
DC: Dice coefficient
GLCM: Grey level co-occurrence matrix
GLRLM: Grey level run length matrix
HD: Hausdorff distance
IBEX: Imaging Biomarker Explorer
IBSI: Image Biomarker Standardisation Initiative
ICC: Intraclass correlation coefficient
IQR: Interquartile range
MRC: Mean relative change
RFs: Radiomic features
ROI: Region of interest
